# Supplementary material for: Association between various cathepsins and uterine leiomyoma: A Mendelian randomization analysis
Source: PLoS One. 2024 Sep 12;19(9):e0310292. doi: 10.1371/journal.pone.0310292 (PMC11392342; doi:10.1371/journal.pone.0310292)
Supplement: S1 Table — (DOCX) [file pone.0310292.s001.docx]

## Supplementary Material 1 Table: The SNPs data of various cathepsins and UL.

**S1 Table A The SNPs data of CTSB.** Among the CTSB SNPs, rs150370599 include a trait of cathepsin L1 measurement, rs1692819 include a trait of cathepsin B measurement. PMID: 29875488. URL: https://gwas.mrcieu.ac.uk/datasets/prot-a-718/

| **CTSB-SNP** | **Chr:BP** | **Effect allele** | **Other allele** | **MAF** | **P-value** | **F-stat** |
| --- | --- | --- | --- | --- | --- | --- |
| rs113646963 | 2:204530789 | T | C | 0.09708 | 2.95E-06 | 21.8507 |
| rs11563944 | 7:89816334 | G | A | 0.26799 | 3.55E-06 | 21.56567 |
| rs117345475 | 11:131588071 | G | A | 0.02062 | 3.74E-06 | 21.55826 |
| rs117486267 | 15:25484738 | C | T | 0.02468 | 1.58E-06 | 23.0377 |
| rs13152767 | 4:122367502 | A | G | 0.02617 | 1.62E-06 | 23.00016 |
| rs143557119 | 11:116385842 | A | G | 0.02199 | 4.79E-06 | 20.92144 |
| rs147881440 | 17:80969815 | A | G | 0.06428 | 4.79E-06 | 20.89208 |
| rs148930853 | 8:114408661 | C | T | 0.01211 | 3.02E-06 | 21.81467 |
| rs150370599 | 11:60718792 | T | C | 0.08341 | 1.78E-06 | 22.81787 |
| rs150811995 | 10:38633385 | C | A | 0.01878 | 4.57E-06 | 21.01912 |
| rs1692819 | 8:38633385 | A | G | 0.29182 | 5.25E-54 | 240.1369 |
| rs36021960 | 7:47111670 | A | G | 0.12637 | 3.02E-06 | 21.81939 |
| rs7249773 | 19:18289760 | A | G | 0.15121 | 6.03E-07 | 24.82935 |
| rs72863882 | 10:134275165 | A | G | 0.08353 | 6.46E-08 | 29.25102 |
| rs75773690 | 1:24464363 | A | G | 0.02192 | 4.17E-06 | 21.19007 |
| rs76089522 | 3:140389189 | A | G | 0.10475 | 2.88E-06 | 21.91841 |
| rs9905973 | 17:26735142 | A | G | 0.271 | 3.55E-07 | 25.914 |

**S1 Table B The SNPs data of CTSE** PMID: 29875488. URL: https://gwas.mrcieu.ac.uk/datasets/prot-a-720/

| **CTSE-SNP** | **Chr:BP** | **Effect allele** | **Other allele** | **MAF** | **P-value** | **F-stat** |
| --- | --- | --- | --- | --- | --- | --- |
| rs10900907 | 6:1448387 | A | G | 0.4897 | 3.23594E-06 | 21.70463668 |
| rs112918835 | 8:109846123 | T | C | 0.0413 | 3.71535E-06 | 21.41639455 |
| rs13089837 | 3:5094952 | C | T | 0.4663 | 4.16869E-06 | 21.233664 |
| rs189009983 | 4:102582604 | T | C | 0.02279 | 4.67735E-06 | 20.9598784 |
| rs1936839 | 10:129433747 | C | G | 0.2237 | 1.99526E-06 | 22.54084866 |
| rs57135345 | 10:26671589 | T | C | 0.08244 | 2.95121E-06 | 21.87546806 |
| rs57689619 | 4:189431921 | G | A | 0.09318 | 2.81838E-06 | 21.90408652 |
| rs74677283 | 11:19486084 | T | C | 0.06486 | 6.76083E-07 | 24.64976142 |
| rs8066936 | 17:754600 | A | G | 0.1809 | 4.2658E-06 | 21.07516213 |
| rs150811995 | 10:38633385 | C | A | 0.01878 | 4.57E-06 | 21.01912 |

**S1 Table C The SNPs data of CTSF** PMID: 29875488. URL: https://gwas.mrcieu.ac.uk/datasets/prot-a-722/

| **CTSF-SNP** | **Chr:BP** | **Effect allele** | **Other allele** | **MAF** | **P-value** | **F-stat** |
| --- | --- | --- | --- | --- | --- | --- |
| rs10745925 | 12:102218899 | C | T | 0.29402 | 9.12E-27 | 9.93079E+50 |
| rs112526544 | 7:26300072 | G | A | 0.02831 | 0.000000692 | 3.01706E+11 |
| rs115901379 | 2:106335628 | G | T | 0.02042 | 0.00000339 | 16134245264 |
| rs1260326 | 2:27730940 | C | T | 0.59849 | 6.92E-11 | 5.63033E+18 |
| rs143015877 | 13:23327148 | T | C | 0.25734 | 0.00000398 | 1108321128 |
| rs1791679 | 11:66337874 | A | C | 0.28947 | 2.51E-18 | 8.75828E+33 |
| rs183683891 | 6:133102456 | T | C | 0.01536 | 0.00000302 | 30128682953 |
| rs186369051 | 5:93830000 | T | C | 0.01504 | 0.00000263 | 33560010988 |
| rs61866943 | 10:85428041 | G | T | 0.01874 | 0.000000912 | 2.96858E+11 |
| rs647400 | 1:78649146 | A | G | 0.19253 | 0.00000204 | 5349058055 |
| rs7564167 | 2:645190 | G | A | 0.93594 | 0.00000049 | 2.92262E+11 |

**S1 Table D The SNPs data of CTSG** PMID: 29875488. URL: https://gwas.mrcieu.ac.uk/datasets/prot-a-723/

| **CTSG-SNP** | **Chr:BP** | **Effect allele** | **Other allele** | **MAF** | **P-value** | **F-stat** |
| --- | --- | --- | --- | --- | --- | --- |
| rs10170044 | 2:171303727 | G | A | 0.39112 | 4.7863E-06 | 20.99173029 |
| rs114418234 | 2:155060275 | C | A | 0.03719 | 2.23872E-06 | 22.40929941 |
| rs116142041 | 4:9787836 | A | G | 0.03645 | 4.57088E-07 | 25.46833852 |
| rs117133380 | 14:107206586 | A | C | 0.01556 | 2.63027E-06 | 22.05911878 |
| rs147099093 | 3:37730576 | A | G | 0.02053 | 1.38038E-06 | 23.29016694 |
| rs192289769 | 3:134273074 | G | C | 0.01364 | 5.62341E-07 | 25.02745493 |
| rs35241999 | 12:11184504 | G | A | 0.02374 | 2.95121E-06 | 21.81717673 |
| rs4702448 | 5:7295725 | A | C | 0.66583 | 1.44544E-06 | 23.1488074 |
| rs497459 | 7:49838522 | C | T | 0.86534 | 1.94984E-06 | 22.66587902 |
| rs56284011 | 17:43079322 | C | G | 0.03943 | 1.7378E-06 | 22.85306365 |
| rs62493038 | 8:12993071 | T | C | 0.03503 | 2.88403E-06 | 21.87658692 |
| rs72773561 | 10:9677794 | C | T | 0.05703 | 1.69824E-06 | 22.94192278 |
| rs77893942 | 8:69507645 | A | T | 0.03128 | 8.70964E-07 | 24.22082728 |

**S1 Table E The SNPs data of CTSH** PMID: 29875488. URL: https://gwas.mrcieu.ac.uk/datasets/prot-a-725/

| **CTSH-SNP** | **Chr:BP** | **Effect allele** | **Other allele** | **MAF** | **P-value** | **F-stat** |
| --- | --- | --- | --- | --- | --- | --- |
| rs12911554 | 15:51542757 | T | C | 0.55027 | 1.8197E-06 | 22.78065428 |
| rs146037740 | 18:11507434 | A | G | 0.02292 | 3.46737E-07 | 25.98914304 |
| rs147991203 | 4:140256765 | T | C | 0.02671 | 1.1749E-06 | 23.63494324 |
| rs34593439 | 15:79234957 | A | G | 0.11066 | 1E-200 | 1098.941662 |
| rs35628511 | 1:24667702 | T | C | 0.3116 | 4.16869E-06 | 21.22092615 |
| rs508807 | 7:152771726 | C | G | 0.81025 | 2.39883E-06 | 22.29610352 |
| rs60018174 | 16:5452751 | T | C | 0.1159 | 7.24436E-07 | 24.52130107 |
| rs62013235 | 15:79246166 | A | G | 0.091 | 1.34896E-20 | 86.5933642 |
| rs62474230 | 8:3347249 | C | G | 0.06195 | 2.69153E-06 | 22.06771313 |
| rs74342103 | 19:36003934 | T | A | 0.02583 | 3.46737E-06 | 21.51040428 |

**S1 Table F The SNPs data of CTSO** PMID: 29875488. URL: https://gwas.mrcieu.ac.uk/datasets/prot-a-726/

| **CTSO-SNP** | **Chr:BP** | **Effect allele** | **Other allele** | **MAF** | **P-value** | **F-stat** |
| --- | --- | --- | --- | --- | --- | --- |
| rs10902420 | 12:132150183 | G | A | 0.89909 | 1.23027E-06 | 23.56293403 |
| rs146963690 | 2:156493388 | G | T | 0.0101 | 2.29087E-06 | 22.3224084 |
| rs149159018 | 3:132077351 | G | A | 0.01105 | 1.54882E-07 | 27.55466474 |
| rs17288007 | 4:159878046 | G | A | 0.06381 | 2.45471E-06 | 22.22190458 |
| rs181844705 | 5:75333560 | G | A | 0.02318 | 1.09648E-06 | 23.7669717 |
| rs1870736 | 15:88624621 | G | C | 0.42746 | 9.33254E-07 | 24.08483734 |
| rs2439803 | 11:129083967 | G | A | 0.15304 | 5.12861E-07 | 25.2284397 |
| rs4076941 | 11:11261567 | C | T | 0.36872 | 2.13796E-06 | 22.58063341 |
| rs4297371 | 10:11249367 | C | A | 0.79306 | 3.54813E-06 | 21.54976574 |
| rs4843804 | 16:88156187 | A | G | 0.0336 | 3.46737E-06 | 21.54725354 |
| rs7140599 | 14:39942467 | C | T | 0.57698 | 1.1749E-06 | 23.58630815 |
| rs78943701 | 10:73736674 | A | G | 0.01536 | 2.95121E-06 | 21.83852469 |
| rs9932172 | 16:7642591 | T | C | 0.10769 | 1.7378E-06 | 22.80598379 |

**S1 Table G The SNPs data of CTSS** PMID: 29875488. URL: https://gwas.mrcieu.ac.uk/datasets/prot-a-727/

| **CTSS-SNP** | **Chr:BP** | **Effect allele** | **Other allele** | **MAF** | **P-value** | **F-stat** |
| --- | --- | --- | --- | --- | --- | --- |
| rs1022239 | 13:65424046 | T | A | 0.58887 | 3.71535E-06 | 21.418384 |
| rs10516855 | 4:90924478 | C | T | 0.09552 | 2.95121E-06 | 21.87395129 |
| rs1060435 | 11:68855595 | G | A | 0.39793 | 2.75423E-07 | 26.45483405 |
| rs113108135 | 5:166659513 | C | G | 0.06933 | 1.69824E-06 | 22.88774727 |
| rs116623438 | 5:102689283 | C | T | 0.02023 | 9.12011E-07 | 24.0950981 |
| rs118010753 | 21:18833920 | C | T | 0.01437 | 3.46737E-06 | 21.54814682 |
| rs12804405 | 11:24422062 | A | G | 0.01019 | 2.0893E-06 | 22.50730124 |
| rs13150189 | 4:181239467 | A | G | 0.21113 | 3.46737E-07 | 25.85689265 |
| rs13196989 | 6:239373 | T | C | 0.13099 | 2.5704E-06 | 22.09502702 |
| rs13212873 | 6:143059208 | C | T | 0.0116 | 3.54813E-06 | 21.47569874 |
| rs13411643 | 2:60351132 | C | T | 0.13712 | 5.01187E-07 | 25.30311104 |
| rs2470994 | 7:47430626 | C | T | 0.28088 | 3.38844E-06 | 21.53488431 |
| rs41271951 | 1:150737220 | G | A | 0.08349 | 7.07946E-94 | 421.7680749 |
| rs4313886 | 18:74370742 | C | T | 0.13446 | 3.89045E-06 | 21.36212423 |
| rs4581957 | 2:174866439 | A | G | 0.13414 | 1.14815E-06 | 23.64890223 |
| rs529565 | 9:136149500 | C | T | 0.31269 | 7.76247E-07 | 24.43927017 |
| rs61870690 | 10:127523966 | G | A | 0.04143 | 7.24436E-07 | 24.50539482 |
| rs6657328 | 1:107433954 | C | G | 0.7184 | 4.89779E-06 | 20.81036097 |
| rs73099998 | 12:58633987 | T | C | 0.20811 | 2.18776E-06 | 22.47006241 |
| rs74804137 | 12:18017954 | C | T | 0.01757 | 1.58489E-07 | 27.52262082 |
| rs7614425 | 3:98174258 | A | G | 0.09254 | 1.8197E-06 | 22.80281542 |
| rs77792819 | 12:94386245 | G | A | 0.04096 | 1.94984E-06 | 22.66681849 |
| rs78767885 | 19:18112020 | C | T | 0.08725 | 2.69153E-06 | 22.0132115 |
| rs989576 | 4:127182592 | T | C | 0.29843 | 2.5704E-06 | 22.10352725 |

**S1 Table H The SNPs data of CTSL2** PMID: 29875488. URL: https://gwas.mrcieu.ac.uk/datasets/prot-a-728/

| **CTSL2-SNP** | **Chr:BP** | **Effect allele** | **Other allele** | **MAF** | **P-value** | **F-stat** |
| --- | --- | --- | --- | --- | --- | --- |
| rs10817163 | 9:99902524 | A | T | 0.22045 | 7.07946E-11 | 42.54987232 |
| rs114113108 | 1:75257391 | C | G | 0.0129 | 4.36516E-07 | 25.53180876 |
| rs116407656 | 4:68099087 | C | T | 0.02439 | 2.75423E-06 | 21.98908747 |
| rs117714361 | 12:109142132 | G | A | 0.02685 | 4.89779E-06 | 20.84961227 |
| rs13068566 | 3:102862667 | G | A | 0.46617 | 2.88403E-06 | 21.94182825 |
| rs148608463 | 12:121413027 | A | G | 0.35415 | 4.57088E-06 | 21.00991688 |
| rs151179824 | 3:173226009 | A | G | 0.02158 | 2.34423E-06 | 22.25878354 |
| rs1523319 | 3:2292279 | G | C | 0.28541 | 3.54813E-06 | 21.44453437 |
| rs2302837 | 17:6014176 | G | A | 0.92494 | 3.31131E-07 | 26.03458412 |
| rs7669728 | 4:146891101 | C | T | 0.43633 | 3.0903E-07 | 26.23536797 |
| rs7898416 | 10:83700161 | G | A | 0.0705 | 3.23594E-06 | 21.68244947 |

**S1 Table I The SNPs data of CTSZ** PMID: 29875488. URL: https://gwas.mrcieu.ac.uk/datasets/prot-a-729/

| **CTSZ-SNP** | **Chr:BP** | **Effect allele** | **Other allele** | **MAF** | **P-value** | **F-stat** |
| --- | --- | --- | --- | --- | --- | --- |
| rs10745925 | 12:102218899 | C | T | 0.29402 | 2.29087E-42 | 185.615015 |
| rs10761760 | 10:65179152 | C | A | 0.44944 | 3.0903E-07 | 26.21598762 |
| rs1135945 | 4:516586 | A | G | 0.20839 | 4.16869E-06 | 21.17222768 |
| rs114675081 | 3:106826623 | G | A | 0.01467 | 1.65959E-06 | 22.97753077 |
| rs116920068 | 10:54555095 | A | G | 0.0289 | 3.01995E-06 | 21.81101994 |
| rs148201372 | 3:32683740 | T | A | 0.01546 | 1.04713E-06 | 23.81036028 |
| rs148370779 | 20:57602212 | T | C | 0.01316 | 2.5704E-57 | 254.6118417 |
| rs298724 | 9:94023321 | C | T | 0.05507 | 3.98107E-06 | 21.28330178 |
| rs36128387 | 1:235460258 | T | C | 0.02261 | 1.54882E-06 | 23.06941858 |
| rs4761709 | 12:93785170 | G | A | 0.40882 | 0.000001 | 23.92742053 |
| rs67845377 | 19:19275802 | T | C | 0.13855 | 3.71535E-06 | 21.40979366 |
| rs7656806 | 4:138271322 | C | A | 0.51592 | 1.86209E-07 | 27.1249646 |

**S1 Table J The SNPs data of UL** PMID: 36726022. URL: https://gwas.mrcieu.ac.uk/datasets/finn-b-CD2_BENIGN_LEIOMYOMA_UTERI/

| **UL-SNP** | **Chr:BP** | **Effect allele** | **Other allele** | **MAF** | **P-value** | **F-stat** |
| --- | --- | --- | --- | --- | --- | --- |
| rs10737892 | 1:244377774 | T | G | 0.6682 | 1.337E-08 | 32.27923182 |
| rs11245981 | 11:18605 | A | G | 0.07804 | 1.08201E-08 | 32.65306122 |
| rs115396598 | 4:54264117 | A | G | 0.002051 | 6.44466E-11 | 42.67153138 |
| rs11685032 | 2:11674295 | C | A | 0.6626 | 1.17501E-08 | 32.67743373 |
| rs117245733 | 13:40723944 | A | G | 0.03044 | 2.93427E-32 | 139.9464608 |
| rs1220703 | 4:70712161 | T | A | 0.6287 | 2.58583E-12 | 48.68233471 |
| rs12213593 | 6:152896250 | A | T | 0.2423 | 3.74404E-09 | 34.873813 |
| rs13209922 | 6:36716417 | T | C | 0.1665 | 1.43999E-08 | 32.24380151 |
| rs146661435 | 10:105277987 | AT | A | 0.09153 | 3.66902E-10 | 39.42934827 |
| rs17880096 | 22:29105202 | C | G | 0.0652 | 2.34801E-09 | 35.5809929 |
| rs2207549 | 11:32368457 | T | A | 0.3487 | 2.57988E-22 | 93.73108368 |
| rs2242652 | 5:1280028 | A | G | 0.23 | 9.78814E-24 | 101.3288891 |
| rs2553773 | 11:35083633 | G | C | 0.5606 | 4.81948E-12 | 47.80426025 |
| rs3744399 | 17:7154582 | C | T | 0.1017 | 2.58202E-08 | 30.95788504 |
| rs3744962 | 18:674320 | G | A | 0.125 | 8.36296E-09 | 33.30251736 |
| rs3804984 | 3:4716214 | C | T | 0.4684 | 4.427E-10 | 38.77008057 |
| rs41308088 | 20:62293118 | T | C | 0.08846 | 1.093E-08 | 32.7184 |
| rs58415480 | 6:152562271 | G | C | 0.2315 | 3.07326E-53 | 237.4032202 |
| rs6001799 | 22:40539200 | A | G | 0.256 | 1.15107E-13 | 54.92231188 |
| rs61768001 | 1:22465820 | C | T | 0.157 | 1.69083E-23 | 100.3451249 |
| rs62323678 | 4:54541340 | A | T | 0.04696 | 4.36999E-09 | 34.4569 |
| rs7128746 | 11:112588144 | C | T | 0.4701 | 2.04498E-08 | 31.29003906 |
| rs7250783 | 19:13376536 | A | G | 0.3379 | 6.528E-09 | 33.46836763 |
| rs73006241 | 11:108089151 | C | T | 0.225 | 5.31986E-14 | 56.49536503 |
| rs7334326 | 13:50967732 | C | G | 0.1545 | 3.95904E-08 | 30.18753228 |
| rs78378222 | 17:7571752 | G | T | 0.0173 | 7.27947E-69 | 307.8864214 |
| rs8048853 | 16:50095595 | T | C | 0.1813 | 6.3841E-12 | 47.4846281 |
| rs9419958 | 10:105675946 | C | T | 0.8835 | 1.81899E-08 | 31.74779046 |
| rs9811216 | 3:169487501 | C | T | 0.2842 | 1.45499E-10 | 41.28746039 |

**S1 Table K The SNPs data of UL (all cancers excluded)**

PMID: 36726022. URL:https://gwas.mrcieu.ac.uk/datasets/finn-b-CD2_BENIGN_LEIOMYOMA_UTERI_EXALLC/

| **ULEX-SNP** | **Chr:BP** | **Effect allele** | **Other allele** | **MAF** | **P-value** | **F-stat** |
| --- | --- | --- | --- | --- | --- | --- |
| rs11089974 | 22:40543608 | T | C | 0.2509 | 8.02E-13 | 51.12717331 |
| rs11245981 | 11:186059 | A | G | 0.07771 | 1.98E-08 | 31.54996423 |
| rs115396598 | 4:54264117 | A | G | 0.002094 | 3.66E-10 | 39.31357059 |
| rs11692588 | 2:11684484 | G | A | 0.6819 | 1.67E-08 | 31.89863122 |
| rs117245733 | 13:40723944 | A | G | 0.03091 | 5.12E-30 | 129.547865 |
| rs12038474 | 1:22403357 | A | G | 0.1926 | 1.74E-22 | 95.61794973 |
| rs1220703 | 4:70712161 | T | A | 0.6304 | 2.5E-10 | 40.23448239 |
| rs12213593 | 6:152896250 | A | T | 0.2428 | 9.97E-09 | 32.95058189 |
| rs12638862 | 3:169477506 | G | A | 0.2825 | 2.32E-11 | 44.47447955 |
| rs12865518 | 13:41143190 | A | G | 0.2302 | 4.07E-08 | 29.97215991 |
| rs146661435 | 10:105277987 | AT | A | 0.09181 | 5.07E-10 | 38.64425329 |
| rs17880096 | 22:29105202 | C | G | 0.06493 | 1.5E-10 | 41.14169358 |
| rs2207549 | 11:32368457 | T | A | 0.3492 | 4.87E-21 | 88.62877551 |
| rs2553773 | 11:35083633 | G | C | 0.5614 | 4.49E-11 | 43.67917915 |
| rs3804984 | 3:4716214 | C | T | 0.4681 | 2.44E-10 | 40.2699983 |
| rs58415480 | 6:152562271 | G | C | 0.2329 | 1.28E-50 | 224.0516344 |
| rs61913672 | 11:107983096 | T | C | 0.2247 | 6.46E-15 | 60.74253906 |
| rs62033029 | 16:50107273 | A | G | 0.1808 | 5.75E-11 | 42.73985098 |
| rs62235753 | 22:29272637 | T | C | 0.01145 | 8.33E-11 | 42.16723802 |
| rs62323678 | 4:54541340 | A | T | 0.04713 | 1.08E-08 | 32.6216716 |
| rs7250783 | 19:13376536 | A | G | 0.3378 | 6.45E-09 | 33.88903061 |
| rs75691080 | 20:62269750 | T | C | 0.1139 | 2.31E-09 | 35.60111111 |
| rs77234976 | 17:7141140 | G | C | 0.1078 | 1.39E-08 | 32.09354246 |
| rs9419958 | 10:105675946 | C | T | 0.8836 | 1.41E-09 | 36.58488076 |

**S1 Table L The SNPs’ URL of UL (independent datasets)**

| **UL-independent datasets’ GWAS ID** | **URL** |
| --- | --- |
| ukb-b-9536 | <https://gwas.mrcieu.ac.uk/datasets/ukb-b-9536/> |
| ukb-b-6528 | <https://gwas.mrcieu.ac.uk/datasets/ukb-b-6528/> |
| ukb-a-522 | <https://gwas.mrcieu.ac.uk/datasets/ukb-a-522/> |
| ukb-e-D25 CSA | <https://gwas.mrcieu.ac.uk/datasets/ukb-e-D25_CSA/> |
