# Supplementary material for: Association between various cathepsins and uterine leiomyoma: A Mendelian randomization analysis
Source: PLoS One. 2024 Sep 12;19(9):e0310292. doi: 10.1371/journal.pone.0310292 (PMC11392342; doi:10.1371/journal.pone.0310292)
Supplement: S1 Fig — (DOCX) [file pone.0310292.s002.docx]

**Supplementary Material 2 Figure: Forest plot.** Forest plot of the MR analysis of the causal relationship between CTSB and UL. For the effect of CTSB on UL, 6 SNPs were excluded because of leave-one-out analysis (rs13152767, rs143557119, rs117486267, rs1692819, rs148930853, rs72863882).

**
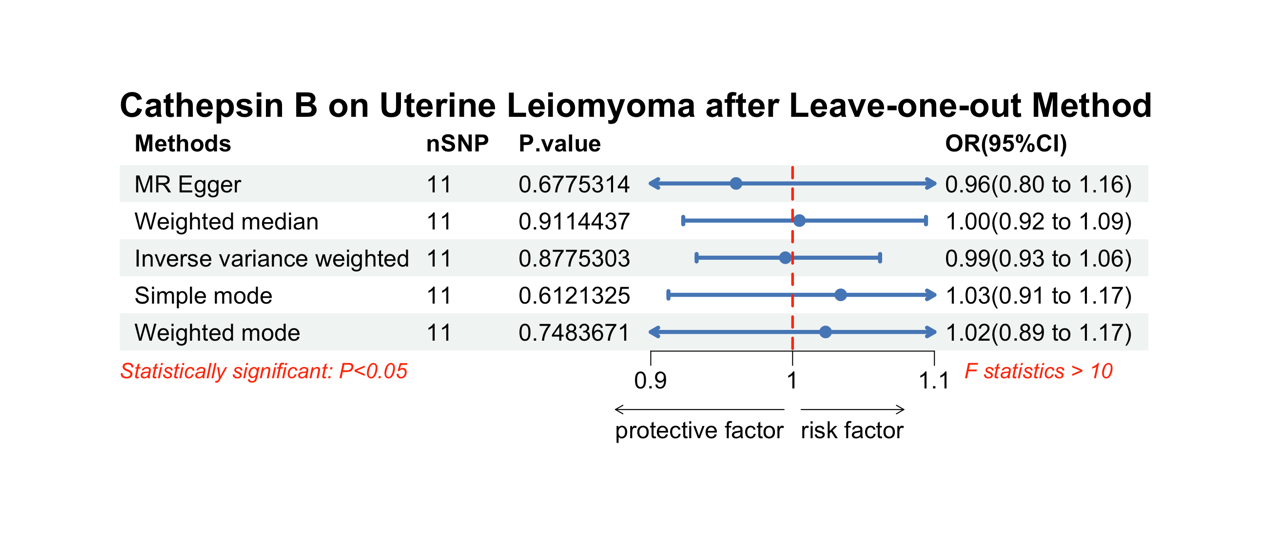
**
