# Supplementary material for: Association between various cathepsins and uterine leiomyoma: A Mendelian randomization analysis
Source: PLoS One. 2024 Sep 12;19(9):e0310292. doi: 10.1371/journal.pone.0310292 (PMC11392342; doi:10.1371/journal.pone.0310292)

**Supplementary Material 3 Figure:** **Scatter plot.** Scatter plot of the MR analysis of the causal relationship between CTSB on UL(all cancers excluded).


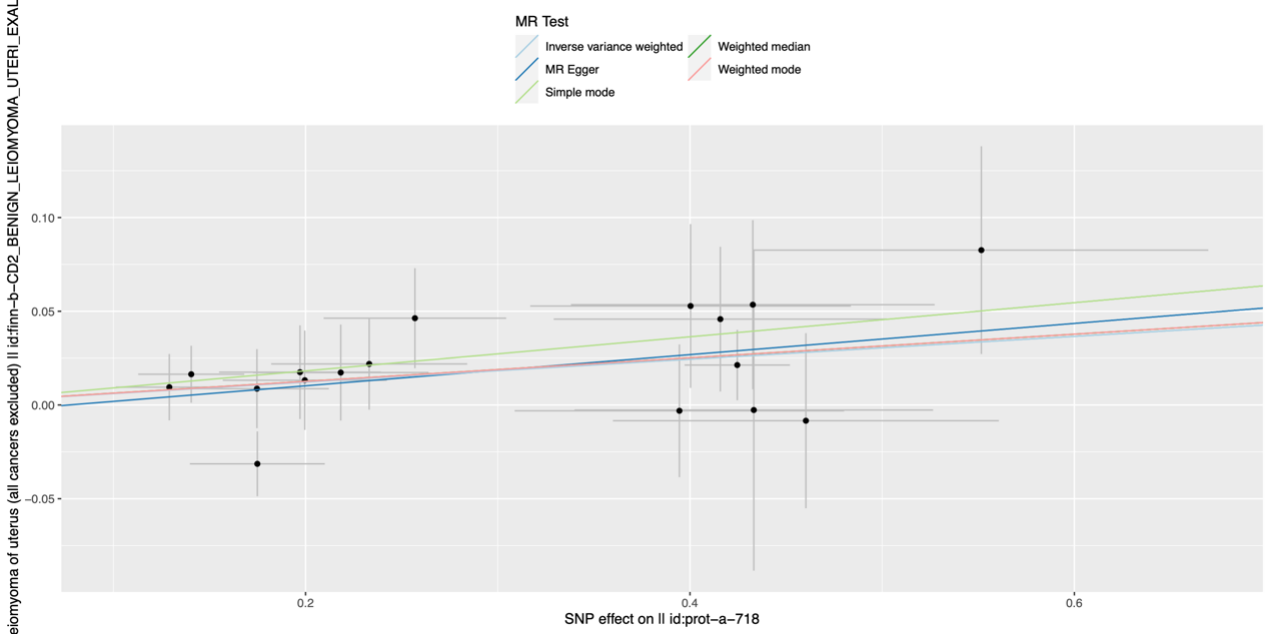

Supplement: S2 Fig — (DOCX) [file pone.0310292.s003.docx]
