# Supplementary material for: Association between various cathepsins and uterine leiomyoma: A Mendelian randomization analysis
Source: PLoS One. 2024 Sep 12;19(9):e0310292. doi: 10.1371/journal.pone.0310292 (PMC11392342; doi:10.1371/journal.pone.0310292)
Supplement: S3 Fig — (DOCX) [file pone.0310292.s004.docx]

**Supplementary Material 4 Figure:** **Leave-one-out plot of CTSB on UL (all cancers excluded).**Leave-one-out plot of the MR analysis of the causal relationship between CTSB on UL(all cancers excluded). Each segment represents the remaining MR analysis results when the current SNP is removed, and it can be observed that the results are still statistically significant when any SNP is removed.

**
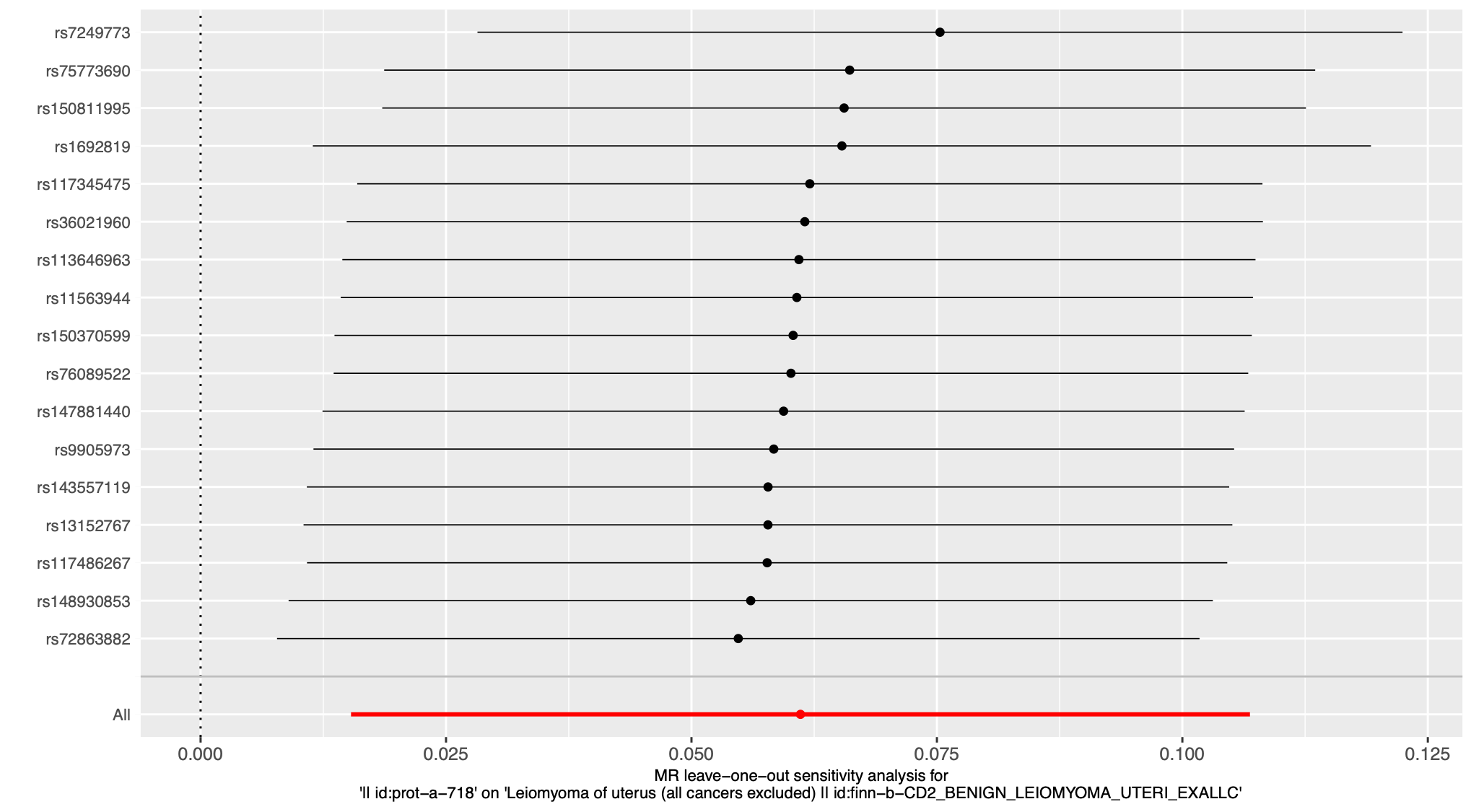
**
