# Supplementary material for: Association between various cathepsins and uterine leiomyoma: A Mendelian randomization analysis
Source: PLoS One. 2024 Sep 12;19(9):e0310292. doi: 10.1371/journal.pone.0310292 (PMC11392342; doi:10.1371/journal.pone.0310292)
Supplement: Attachment — Submitted filename: Response to Reviewers.pdf [file pone.0310292.s005.pdf]

Dear Editor and Reviewers,

The authors feel great thanks for the editor and all reviewers for their professional review work on our article “Bidirectional mendelian randomization analyses explore the relationship between various cathepsins and uterine leiomyoma: Cathepsin B is the risk factor of uterine leiomyoma” (Submission ID: PONE-D-24-11417). We gratefully appreciate the valuable comments and suggestions provided by the reviewers, and we believe that their input has greatly improved our manuscript.

We have carefully considered the reviewers’ suggestions and have made comments and revisions to address all their concerns. According to your suggestions, The reviewer comments are laid out below in normoal font and specific concerns have been numbered. Our responses to the editor’s and reviewers’ comments are noted below (marked with ‘>>Reply:’ and highlighted in blue) . Quote texts from the manuscript are given in *purple and italic*. Any corrections in the manuscript are red highlighted.

In general, after supplementing the multiple tests required by the reviewers, we found that our results lacked certain persuasivity. In order to present the results more rigorously, we have added a significant amount of content and modified the way we describe the results for readers. We hope this revised version is acceptable for publication.

On behalf of all authors,

Tingxiu Liu & Yuehan Ren

Corrsponding Author:

* Xinmin Liu
Email: XinminLIU9626@126.com
MD., Ph.D., Professor
Guang’anmen Hospital, China Academy of Chinese Medical Sciences, No. 5, Beixiange, Xicheng District, Beijing, China

* Peiyu Yan
Email: teamzhang@126.com
MD., Ph.D., Professor
Faculty of Chinese Medicine, Macau University of Science and Technology,
State Key Laboratory of Quality Research in Chinese Medicines, Macau University of Science and Technology Zhuhai MUST Science and Technology Research Institute, Macau University of Science and Technology, Macao, Macao SAR, China.

Journal Requirements and Editor Comments:

Reviewer #1:

Summary:

Cause for major concern:

Line 175: "Further MR analysis was performed on the subgroup of UL (excluding all cancers)"– please specify how this subgroup was identified. This is a major concern as I do not see a phenotype for UL (excluding all cancers) in the public FinnGen GWAS data release. I am only able to see GWAS results for three uterine cancers – carcinoma in situ of cervix uteri, malignant neoplasm of corpus uteri, and malignant neoplasm of cervix uteri, where the controls exclude all cancers. So please provide:

1. The FinnGen endpoint code (for example for UL it is: CD2_BENIGN_LEIOMYOMA_UTERI),

2. Number of cases and controls

3. An explanation from the FinnGen database of how this subgroup of UL (excluding all cancers) was selected. As this is the analysis with significant results, it is not possible to interpret the results presented without this information.

>>Reply:

Thank you for pointing out the missing information, when we decided to do the UL (excluding all cancers) group, we had checked the FinnGen endpoint code, but the "finn-b-CD2_BENIGN_LEIOMYOMA_UTERI_EXALLC" in IEU OPEN GWAS wasn't shown in FinnGen. Then we sent an email to finngen-endpoints@helsinki.fi. According to the response from Dr Mervi Aavikko, Associate Professor, FinnGen subjects are still assigned to these phenotypic endpoints by Endpointter, including the systematic control selection for cancer endpoints, such as EXALLC version selected for malignant cancers (C3_) and EXALLC version omitted for benign cancers (CD2_BENIGN_).

The dataset "finn-b-CD2_BENIGN_LEIOMYOMA_UTERI_EXALLC" is still available on the IEU OPEN GWAS website for MR analysis.

To provide more information, we have included the table of CTSB on UL (excluding all cancers) and replenish a table of SNPs' traits in the S1 Table1.

The dataset is a control group, while the patients carried UL but all cancers were

excluded.

Ncase=18,060

Ncontrol=88,982

Leiomyoma of uterus (all cancers excluded)

Dataset: finn-b-CD2_BENIGN_LEIOMYOMA_UTERI_EXALLC

**Year**	2021
**Category**	Binary
**Sub category**	NA
**Population**	European
**Sex**	Males and Females
**ncase**	18,060
**ncontrol**	88,982
**Number of SNPs**	16,379,176
**Unit**	NA
**Author**	NA
**Consortium**	NA
**Ontology**	NA
**Build**	HG19/GRCh37
**Note**	CD2_BENIGN_LEIOMYOMA_UTERI_EXALLC

Top 30 related datasets
N/A

Other Major Comments:

1. The Introduction requires more references, for example: line 74, line 80, line 84, etc. that explain the prevalence, aetiology, and risk factors for UL.

>>Reply:

We sincerely appreciate the valuable comments. We have checked the literature carefully and added more references into Introduction session in the revised manuscript.

The references of the prevelance, aetiology, and risk factors for UL are as follows (line 55, line 56, line 61):

(a) Prevelance: However, these data alone may not be sufficiently accurate for UL[3]

(b) Aetiology: Studies of family aggregation, prevalence, incidence, and racial differences have shown that genetic factors influence the risk of developing UL[5].

(c) Risk factors: Risk factors for ULs include age, race, hormonal influences, reproductive history, genetic predisposition, lifestyle factors such as physical activity

and diet, and exposure to endocrine disruptors and obesity[4].

2. Line 110: Please implement Steiger filtering, which is available in the TwoSampleMR package, to orient the causal relationship between cathepsins and UL. This can be in addition to the reciprocal MR results presented, but is necessary to ensure that instrument SNPs have stronger effects on the exposure than on the outcome. Ref: https://journals.plos.org/plosgenetics/article?id=10.1371/journal.pgen.1007081

>>Reply:

Thank you for the valuable comments. The MR Steiger method improves statistical efficacy by assessing exposure and outcome variables across samples. We took the recommendation to include the MR Steiger method from the TwoSample MR package to test the data of CTSB on UL, but the instrumental variables did not pass the MR Steiger test, which may affect the accuracy of causal inference, so we corrected the description of the results of the MR analyses and prompted readers to be cautious (line 136, line 280).

The MR Steiger method was taken by testing the measurement error of the IVs[20].

The IVs of the cathepsins did not pass the MR Steiger test. If multiple MR results were adjusted Bonferroni correction ($P < 0.0028$), then the CTSB' s effect on ULs is insignificant ($P=0.0089$). It could not be determined that CTSB is a direct risk factor for UL, and the effect of CTSB on UL still needs to be further investigated.

3. Line 131: Please explain why such a high genome-wide significance threshold ($P < 5E-6$) was selected, when the standard in the field is $P < 5E-8$. Such a high threshold increases your chance of having spurious SNPs in the instrument, and also may lead to a weak instrument.

Ref: https://www.nature.com/articles/s43586-021-00056-9

>>Reply:

Thank you for your advice, for UL, we have conduct the MR analysis using the standard $P < 5E-8$, the threshold was missing in the draft. For cathepsins, the standard $P < 5E-6$ was used. Several MR studies involved cathepsins were using $P < 5E-6$ as a standard. It is possible for the user to extract the top-hits from this data source using their own criteria. And while the $P < 5E-8$ wasn't suitable for the data, $P < 5E-6$ is still a choice (line 117).

Ref: https://www.ncbi.nlm.nih.gov/pmc/articles/PMC5976434/

All study participants were of European ancestry. SNPs of cathepsins were selected with a threshold of P<5e×10-6 between cathepsin and IVs according to previous studies, and SNPs of UL were selected with a genome-wide significance threshold of P<5e×10-8[17].

4. Line 132: Please explain the identification of independent SNPs in more detail. Did you use the LD-clumping procedure in the TwoSampleMR package? Please specify if so, and also move the distance for clumping (currently on line 131, which says 10,000 kb) to line 132.

>>Reply:

Thank you for your advice.

The selection of independent instrumental variables (IVs) is a key step in Mendelian randomisation analysis. Independent instrumental variables should fulfil the following three main criteria:

Relevance: instrumental variables must be significantly correlated with the independent variable (exposure). In other words, the instrumental variable should be able to explain the variation in the independent variable. This is usually verified by a statistical test, such as an F-test, which ensures that there is a significant association between the instrumental variable and the independent variable.

Independence: The instrumental variable must be independent of the confounders. That is, the instrumental variable should not be correlated with other factors that may affect the independent variable and the dependent variable (outcome). This criterion ensures that the instrumental variable only influences the dependent variable through the independent variable and not through other pathways.

Exclusion Restriction: the effect of the instrumental variable on the dependent variable must be realised exclusively through the independent variable. This means that the instrumental variable cannot directly affect the dependent variable, ensuring that variation in the dependent variable is transmitted only through the independent variable.

By selecting instrumental variables that satisfy the above criteria, the researcher can use Mendelian randomisation analysis to reveal the causal relationship between the independent variable and the dependent variable, thus reducing the influence of confounding factors and reverse causation and obtaining more reliable causal inferences.

We further explain the identification of independent SNPs in line 84.

IVs must correlate significantly with exposure and explain its variation, as confirmed by statistical tests such as the F-test. IVs are not related to other factors that influence both exposure and outcome, thus avoiding bias. The effect of the IV on the outcome is solely through the exposure, with no direct effect.

The LD-clumping procedure was used, and the distance for clumping is moved (line 121).

To account for the effect of linkage disequilibrium (LD) between SNPs, the LD clustering procedure was used (distance>10,000 kb, r2≤0.001) according to the European 1000 Genome Reference Panel.

5. Line 136: What are the "confounding traits" you tested? Please explain list which SNPs were removed due to association with confounders.

>>Reply:

Thank you for your valuable comments. Confounders were removed by filtering out age at menarche, total testosterone, high BMI, blood pressure, long-term smoking, oral contraceptive use. Please note that the PhennoScanner website is currently unavailable. We use FastTraitR according to data from NHGRI-EBI catalogue instead (line 124).

Ref:

https://academic.oup.com/aje/article/153/1/1/107780?login=false

https://www.rbmojournal.com/article/S1472-6483(23)00683-1/abstract

https://www.ebi.ac.uk/gwas/

Then, those associating with the confounders of ULs were excluded manually according to the data from NHGRI-EBI catalogue (https://www.ebi.ac.uk/gwas/), assessing their association with any previously identified confounding traits (P<5e×10−8), assessing their association with any previously identified confounding traits (P<1e×10−5).

6. Line 138: Please briefly outline how MR-PRESSO identifies outliers, i.e. what criteria make a SNP an outlier?

>>Reply:

Thank you for your comments, in the MR-PRESSO technique, the identification of outliers is conducted through an examination of the residuals from the predicted

outcomes leveraging the instrumental variables. This approach utilizes a graphical representation where the predicted outcomes are juxtaposed against their respective instrumental variables, thereby evaluating the uniformity of the observed data points. Outliers are delineated as those data points that exhibit a substantial divergence from the overarching trend evident within the dataset, suggesting potential breaches in the Mendelian randomization assumptions, including but not limited to horizontal pleiotropy or the presence of measurement error.

In MR-PRESSO package, we use the code below to set SignifThreshold = 0.05 in order to identify the outliner.

run_mr_presso(Mydata,NbDistribution = 10000)

mr_presso(BetaOutcome ="beta.outcome", BetaExposure = "beta.exposure", SdOutcome ="se.outcome", SdExposure = "se.exposure", OUTLIERtest = TRUE,DISTORTIONtest = TRUE, data =Mydata, NbDistribution = 10000, SignifThreshold = 0.05)

We have modified the draft in line 129.

We identified outliners by P<0.05 using MR-PRESSO global test (MR-PRESSO package, version 1. 0)

7. Line 140: It is crucial to provide the F-statistics for all the instruments in all the various analyses.

>>Reply:

Thank you for your advice. The F-statistics for all the instruments in all the various analyses were replenished, as well as the F-statistics' threshold (F-statistics > 10) in all the figures (S1 Table, Fig 2-4, S2 Fig).

8. Line 142: It is not correct that "IVW method is the gold standard of MR", it depends on what assumptions are violated. If none are violated, then IVW is the most sensitive method. Please modify. Ref: https://www.ncbi.nlm.nih.gov/pmc/articles/PMC5506233/

>>Reply:

Thank you for your advice, we have modified the draft, see line 136.

IVW is the most sensitive method on what MR assumptions are violated[19].

9. Line 153: Since you are performing 9 MR tests (for all 9 cathepsins), you should

adjust your P-value for multiple testing. Moreover, since you also perform reciprocal MR (another 9 tests), I would recommend Bonferroni correction (significance at $P < 0.05/18$) or for less stringency as the cathepsins are all somewhat correlated, FDR correction. If you apply Bonferroni, then none of the results will be significant.

>>Reply:

Thank you for your comments. Certainly we should add the Bonferroni correction to correct the result. The results didn't pass the Bonferroni test. However, we've added the Bonferroni test result in the draft to indicate that the MR results need serious consideration. Due to the limitations of the code used to create the Figure, we did not add the multi-corrected p-values to the Figures, but rather explained them in the body of the text (line 137, line 280).

As multiple MR analysis were performed, the result should be assessed with Bonferroni correction ($P < 0.05/18$).

If multiple MR results were adjusted Bonferroni correction ($P < 0.0028$), then the CTSB's effect on ULs is insignificant ($P=0.0089$). It could not be determined that CTSB is a direct risk factor for UL, and the effect of CTSB on UL still needs to be further investigated.

10. Line 157: Please specify which SNPs were removed by sensitivity analysis, and which of the various sensitivity analyses you carried out (leave-one-out, heterogeneity tests, MR-PRESSO, etc.) caused this.

>>Reply:

Thank you for your advice.

It is important to provide the SNPs that were culled and for which sensitivity analysis they were culled, sorry we missed this before. With the code in the TwoSampleMR package, we exported and sorted the leave-one-out statistics after exporting the leave-one-out plots.

When performing the leave-one-out sensitivity analysis for CTSB on UL, we found that the following SNPs had a p-value of >0.05, indicating that the causal relationship of CTSB being a risk factor for UL was not valid when they were excluded.

rs13152767

rs143557119

rs117486267

rs1692819

rs148930853

rs72863882

We have also provided the additional information in S2 Fig.

11. Lines 158-160: The leave-one-out analysis should produce 11 plots, as you have 11 SNPs in the instrument and each forest/scatter plot will display results from repeating the MR without one of the SNPs. You have only provided one forest/scatter plot – please check.

>>Reply:

Thank you for your advice. To briefly explain the meaning of the leave-one-out sensitivity analysis image of positive results in the supplementary figure, each black segment represents the significance of the statistical result after excluding the SNP, so the 11 black segments contained all SNPs. The red segment at the bottom of the image represents the overall results for comparison; if the black segment is in a different position on the axe compared to the red segment, then it is necessary to eliminate the SNP (S4 Fig).

12. Lines 167-172: To my understanding, this is just re-stating the same things already reported in lines 158-161 (but the P-value reported is slightly different, 0.878 vs 0.876 – typo?) Remove this section or explain.

>>Reply:

Thank you for pointing the duplicate text, the corrected sentence were shown only once (line 167).

When the MR results of CTSB were analyzed by the retention method, it was found that the statistical difference disappeared after the removal of specific SNPs (OR = 0.99, 95% CI 0.93, 1.06, P= 0.878), suggesting that CTSB had no causal effect on UL when CTSB was an exposure factor (Fig 2).

13. Line 178: "…remaining cathepsins showed no statistical significance." Why is the protective association of Cathepsin O on UL excluding cancers (Figure 3) not discussed? The strength and significance of this association are comparable to CTSB (OR=0.93, P=0.019).

>>Reply:

Thank you for your comments. We have provided additional clarifications to the original text. The protective effect of CTSO on UL is due to a single specific SNP (rs181844705) and cannot be considered statistically significant (line 183)

In addition, the results showed a protective effect (OR = 0.93, 95% CI 0.88, 0.99, P= 0.019) of cathepsin O (CTSO) on ULs (all cancers excluded). Unfortunately, the protective effect is due to a single SNP (rs181844705) and cannot be considered statistically significant.

14. Figure 1:
a. Could the authors name some specific confounders they evaluated (as they mention in line 136)?
b. The "Exposure" box should cite the study from which genetic instruments were derived.
c. Minor comments: what does the symbol represent in the "Instrumental variables" box? In the "Mendelian randomization analysis" box, MR-PRESSO is mis-spelled.

>>Reply:

Thank you for your opinion. We collected confounders associated with exposure and outcome. The confounders include age at menarche, total testosterone, high BMI, blood pressure, long-term smoking, oral contraceptive use. We redrew a new Figure 1 flowchart using Adobe Illustrator.

15. Figures 2, 3, and 4:
a. Include instrument F-statistics in these plots
b. Adjust the P-value for multiple-testing correction

>>Reply:

Thank you for your advice. We performed the F-statistics of all instrumental variables in the Supplementary Material. In Figures 2, 3, and 4 we supplemented F-statistics screening criteria of all SNPs' strength (F-statistics> 10). In the Results session we discribed the adjusted P-values for multiple corrections.

16. Figure 4: Please extend the axes to capture the full 95% CIs as these are not so large as to make the plots meaningless. In the top plot, include at least [0.95, 1.25] and in the bottom plot, [0.75, 1.30].

>>Reply:

Thank you for your advice. The axes of Figure 4 are extended in the new draft.

17. Supp. Table 1: It seems only one SNP for CTSB would pass a genome-wide significant threshold of P<5E-8, so I'm worried that the results are driven by a single SNP. The leave-one-out plots are also missing. You must also provide F-statistics so we can evaluate the strength of the instrument.

>>Reply:

Thank you for your advice. Indeed, only one SNP can pass this screen under the P<5E-8 criterion. Your concern is a necessary. We also found this when conducting our study, so we conducted a search of the available literature and found that the existing studies on the inclusion of histones for MR analysis all adopted the P<5E-6 criterion. To make sure the strength of the SNPS is strong enough, we supplemented the F-statistics (F > 10).

Minor Comments:

1. Please split the Introduction into three separate paragraphs – one on the aetiology of UL, one on cathepsins, and one on Mendelian Randomisation.

>>Reply:

We appreciated the comment and had split the Introduction into three parts: line 47, line 64, line 73.

2. Line 107 & Figure 1 caption: Please correct that you use multiple GWASs as this is two-sample MR, not "a genome-wide association study (GWAS)" which means one single study.

>>Reply:

Thank you for your comment. We've revised what we said in line 89 and Figure 1 (line 101).

This analysis utilizes data derived from multiple genome-wide association studies (GWAS) to unravel the bidirectional interrelations between ULs and various cathepsins.

3. Line 121: Please specify that you construct instruments for cathepsins for forward MR, and instruments for UL for reciprocal MR – it currently reads like you only did the latter.

>>Reply:

Thank you for your advice, we have adjusted the draft making it readable (line 105).

Forward MR analysis was constructed by instruments for cathepsins, and instruments for UL were selected for reciprocal MR (https://gwas.mrcieu.ac.uk/).

4. Line 122: Which data release of FinnGen did you use?

>>Reply:

Thank you for pointing out the missing information. The discription of FinnGen data could be seen in the reply of **Cause for major concern.**

Reviewer #2:

1. In the abstract session, the authors stated that there are "Emerging evidence suggests a tentative association between cathepsins and uterine leiomyoma.", but could the authors give some citations to support this statement?

>>Reply:

Thank you for your advice. Citation was replenished in the introduction session (line 64).

Ref: https://www.ejog.org/article/S0301-2115(14)00669-1/abstract

Emerging evidence suggests a tentative association between cathepsins and uterine leiomyoma[6].

2. Can the authors specify the standard for eliminating the SNPs for the sensitivity analysis and quote studies using similar methodology?

>>Reply:

Thank you for your advice.

In the original manuscript, the sensitivity analysis methods we mentioned included the MR-Egger intercept test, the leave-one-out method, and the Cochran's Q test, which tested for the presence of heterogeneity and horizontal multiplicity of findings.

In addtion, during the revision process, the reviewers also suggested us to add discription about selecting SNPs, such as LD-clumping, Steiger filtration, and other methods, and to write the P-value of the positive results after Bonferroni multiple correction.

To this we have added the following (line 105-146).

Ref:

https://www.ncbi.nlm.nih.gov/pmc/articles/PMC5506233/

https://www.nature.com/articles/s43586-021-00056-9

3. Can the authors verify the findings using another independent cohort?

>>Reply:

Thank you for your advice. To ensure the reliability of the results, other independent GWAS datasets of UL was used.

It was concluded that a bidirectional negative causal relationship between CTSB and ULs could be validated. (GWAS datasets: ukb-b-9536, ukb-b-6528, ukb-a-522, ukb-e-D25 CSA)

Unfortunately, of the available cohorts, only the FinnGen database provided data on ULs (excluding all cancers), and therefore the positive conclusions we obtained could not be replicated.

4. There's a grammar error in line 157-158.

>>Reply:

We were really sorry for our careless mistakes. Thank you for your reminder.

The editorial office also wants to add the following:

1. Albeit a large number of SNPs analysed, please ensure to use a relatively high genome wide significance threshold ie $p < 10^{-8}$ in order to produce an enough significant SNPs.

>>Reply:

Thank you for your advice. Indeed, only one SNP can pass this screen under the P<5E-8 criterion. Your concern is a necessary. We also found this when conducting our study, so we conducted a search of the available literature and found that the existing studies on the inclusion of histones for MR analysis all adopted the P<5E-6 criterion. To make sure the strength of the SNPS is strong enough, we supplemented the F-statistics (F-statistics > 10).

2. To include details on multiple testing correction to justify for an accurate data

analysis.

>>Reply:

Multiple testing correction was used to justify the data. We also added to the "Discussion - Reseach limitations" session.

3. It is necessary to use literature or a tool such as phenoscanner to validate whether the identified SNPs are associated with any other outcome or exposure.

>>Reply:

Thank you for your valuable comments. SNPs were validate by the tool: FastTraitR according the data from NHGRI-EBI Catalogue to make sure they are not associated with other outcome.

Ref: https://www.ebi.ac.uk/gwas/

4. Interpretation and conclusions drawn must be supported by a robust methodology in order to fulfill our publication criteria 3 (https://journals.plos.org/plosone/s/criteria-for-publication).

>>Reply:

Thank you for the notice.

Journal Requirements:

1. Please ensure that your manuscript meets PLOS ONE's style requirements, including those for file naming. The PLOS ONE style templates can be found at

https://journals.plos.org/plosone/s/file?id=wjVg/PLOSOne_formatting_sample_main_body.pdf and https://journals.plos.org/plosone/s/file?id=ba62/PLOSOne_formatting_sample_title_authors_affiliations.pdf

>>Reply:

Thank you. We have downloaded the template and now the manuscript meets the style requirements. We've checked all the file naming according to the templates.

2. We noticed you have some minor occurrence of overlapping text with the following previous publication(s), which needs to be addressed: https://doi.org/10.1017/thg.2023.48(among others). In your revision ensure you cite

all your sources (including your own works), and quote or rephrase any duplicated text outside the methods section. Further consideration is dependent on these concerns being addressed.

>>Reply:

Thank you very much. The overlapping text was addressed (line 107). It should be noted that this Minor Revision has made many changes to the methodology of the article, and the new manuscript is now methodologically improved from the previous MR study completed by Ren et al. All the sources were cited in the new draft.

3. PLOS requires an ORCID iD for the corresponding author in Editorial Manager on papers submitted after December 6th, 2016. Please ensure that you have an ORCID iD and that it is validated in Editorial Manager. To do this, go to 'Update my Information' (in the upper left-hand corner of the main menu), and click on the Fetch/Validate link next to the ORCID field. This will take you to the ORCID site and allow you to create a new iD or authenticate a pre-existing iD in Editorial Manager. Please see the following video for instructions on linking an ORCID iD to your Editorial Manager account: https://www.youtube.com/watch?v=_xcclfuvtxQ

>>Reply:

Thank you for your reminder. The corresponding author's ORCID iD (0009-0003-0395-7289) is validated in Editorial Manager. The ORCID iD of the first author: 0009-0000-5723-6258.

4. Please amend either the title on the online submission form (via Edit Submission) or the title in the manuscript so that they are identical.

>>Reply:

Thank you very much.

5. Please amend the manuscript submission data (via Edit Submission) to include authors Dr. Tingxiu Liu, Dr. Yuehan Ren, Dr. Junning Zhang, Dr. Hechun Yin, Dr. Zheng Zheng, Dr. Mingyue Zhang, Dr. Yan Liao, Dr. Liangliang Yang, Dr. Chang Liu, and Dr. Peiyu Yan.

>>Reply:

Thank you very much. Submission details have been changed.

6. Your ethics statement should only appear in the Methods section of your manuscript. If your ethics statement is written in any section besides the Methods,

please move it to the Methods section and delete it from any other section. Please ensure that your ethics statement is included in your manuscript, as the ethics statement entered into the online submission form will not be published alongside your manuscript.

>>Reply:

Thank you for your advice.

According to the associate editor and reviewers' comments, we have made extensive modifications to our manuscript and Supplemented extra data to make our results convincing. Thank you again for all your positive comments and valuable suggestions to improve the quality of our manuscript!
