# Supplementary material for: Association between various cathepsins and uterine leiomyoma: A Mendelian randomization analysis
Source: PLoS One. 2024 Sep 12;19(9):e0310292. doi: 10.1371/journal.pone.0310292 (PMC11392342; doi:10.1371/journal.pone.0310292)
Supplement: Attachment — Submitted filename: Response to Reviewers-Minor Revision.pdf [file pone.0310292.s006.pdf]

Dear Editor and Reviewers,

The authors feel great thanks again for the editor and all reviewers for their professional review work on our major-revised article “Association between various cathepsins and uterine leiomyoma: A Mendelian randomization analysis” (Submission ID: PONE-D-24-11417).

We gratefully appreciate the valuable comments and suggestions provided by the reviewers. To this revised manuscript, we have carefully considered the reviewers’ suggestions and have made comments and revisions to address all their concerns.

According to your suggestions, The reviewer comments are laid out below in normoal font and specific concerns have been numbered. Our responses to the editor’s and reviewers’ comments are noted below (marked with ‘>>Reply:’ and highlighted in blue) . Quote texts from the manuscript are given in *purple and italic*. Any corrections in the manuscript are red highlighted.

In general, we have carefully added the suggestions of the reviewers, and

On behalf of all authors,

Tingxiu Liu & Yuehan Ren

Corrsponding Author:

* Xinmin Liu
Email: XinminLIU9626@126.com
MD., Ph.D., Professor
Guang’anmen Hospital, China Academy of Chinese Medical Sciences, No. 5, Beixiange, Xicheng District, Beijing, China

* Peiyu Yan
Email: teamzhang@126.com
MD., Ph.D., Professor
Faculty of Chinese Medicine, Macau University of Science and Technology,
State Key Laboratory of Quality Research in Chinese Medicines, Macau University of Science and Technology Zhuhai MUST Science and Technology Research Institute, Macau University of Science and Technology, Macao, Macao SAR, China.

Journal Requirements and Editor Comments:

Journal Requirements:

Please review your reference list to ensure that it is complete and correct. If you have cited papers that have been retracted, please include the rationale for doing so in the manuscript text, or remove these references and replace them with relevant current references. Any changes to the reference list should be mentioned in the rebuttal letter that accompanies your revised manuscript. If you need to cite a retracted article, indicate the article's retracted status in the References list and also include a citation and full reference for the retraction notice.

>>Reply:

Thank you very much. We appreciate yout work and checked the cited papers, instead of replacing the current references, we added the relating informations (URLs) into the supplementary materials.

Reviewer #1：

Summary:
Congratulations to Liu et al. for thoroughly addressing all my comments. My remaining concerns are mostly about the interpretation of results rather than the methodology.

>>Reply:

Thank you very much for your valuable comments. We understand the concerns and have revised the manuscript to interpret our findings. The track changes of this revision are listed below.

Comments:
1. The association of cathepsin B with UL does not remain after:
a. Adjustment for multiple testing, or
b. Steiger filtering
Both of these give me cause for concern, as they indicate that this is likely a spurious association. While the authors mention this in the Limitations, these two major limitations should be presented in the Abstract, which currently has no indication that the result might not be real. Additionally, please make it clear that the presented finding is for cathepsin B on UL "excluding all cancers".

>>Reply:

Thank you for your advice. Now in the revised manuscript, the concerns have been shown in both the Abstract and Limitations sessions. We have also clarified that the result presented is about cathepsin B on UL "excluding all cancers".

Line 38:

Our findings reveal the association between cathepsin B (CTSB) and an elevated risk of developing UL (all cancers excluded) [Inverse Variance Weighted (IVW) method]: OR=1.06, 95%CI [1.02, 1.11], P=0.008895711.

Line 40:

Although the association does not persist after multiple testing or Steiger filtering, this finding adds to our understanding of the causal relationship between CTSB of various cathepsins and UL (all cancers excluded) and may herald new therapeutic avenues for individuals affected by this condition.

2. The authors say they conducted independent validation of their findings using external datasets (in their response to reviewer #2). Why are these results not presented in the manuscript? I understand that they don't have replication of the findings for UL-excluding cancer, but as the UL (not excluding cancer) result is also presented in the manuscript, it is worth showing the replication of this finding.

>>Reply:

Thank you for your suggestions. Though we couldn't replicate the result of UL-excluding cancer, the results of various cathepsins on an independent datasets of UL is worth to be presented.

We described the missing information in both Method and Limitation session. The URLs of the independant datasets were replenished in the Supplementary materials (S1 Table).

Line 151:

Independent GWAS datasets should be used to confirm associations to ensure the reliability of the results.

Line 293:

To ensure the reliability of the results, we used independent GWAS datasets of UL. It was concluded that a bidirectional negative causal relationship between CTSB and ULs could be validated (S1 Table). Unfortunately, of the available cohorts, only the

FinnGen database provided data on ULs (excluding all cancers), and therefore the positive conclusions we obtained could not be replicated.

According to the editor and reviewers' comments, we have made changes to our manuscript and Supporting informations. Thank you again for all your positive comments and valuable suggestions to improve the quality of our manuscript!
